# Genetic Structure and Biodiversity in Wild *Centropomus parallelus* and in Wild and Recently Domesticated *Centropomus undecimallis* Populations

**DOI:** 10.3390/life13071595

**Published:** 2023-07-20

**Authors:** Marcos Edgar Herkenhoff, Miklos Maximiliano Bajay, Carlos André da Veiga Lima-Rosa Costamilan

**Affiliations:** 1 Department of Biochemical and Pharmaceutical Technology, School of Pharmaceutical Sciences, University of São Paulo (USP), Av. Professor Lineu Prestes, 580, São Paulo 05508-000, Brazil; 2 Food Research Center FoRC, University of São Paulo (USP), Av. Professor Lineu Prestes, 580, São Paulo 05508-000, Brazil; 3 Molecular Genetics Laboratory, Center for Higher Education, South Region, Santa Catarina State University, Laguna 88790-000, Brazil; miklos.bajay@udesc.br (M.M.B.); carlos.lima@udesc.br (C.A.d.V.L.-R.C.)

**Keywords:** microsatellites, *Centropomus parallelus*, *Centropomus undecimalis*, Centropomidae, genetic polymorphism

## Abstract

*Centropomus undecimalis* (common snook) and *Centropomus parallelus* (fat snook) have a wide distribution from southern Florida to southern Brazil. Due to their value as a food source, these species have been heavily exploited through predatory fishing, posing a conservation challenge. To assess their genetic diversity and population structure, we used microsatellite markers. Our findings revealed genetic differences among populations of the same species, highlighting the need for targeted conservation efforts. The microsatellite markers proved effective in assessing genetic variability, providing valuable insights for management and conservation. The parameters Ho (observed heterozygosity) and He (expected heterozygosity) were reliable indicators of genetic diversity, and specific loci showed varying allele numbers across populations. Our study contributes to understanding population genetics in these snook species and supports their conservation. Despite not being classified as endangered, genetic differences among populations emphasize the importance of considering population-level characteristics in conservation strategies. This research lays the foundation for future studies and actions aimed at preserving these valuable fish species. In summary, our study demonstrates the significance of microsatellite markers in assessing genetic variability and population structure in common snook and fat snook, informing conservation efforts for these species.

## 1. Introduction

Brazil has a coastal area spanning over 8000 km, accompanied by a vast volume of freshwater, making it one of the largest hydrographic basins globally. With its rich marine biodiversity, the country’s marine fish farming industry is experiencing continual expansion and economic growth [1,2]. In 2018, global fish production generated revenues exceeding USD 260 billion. In Brazil, this sector contributed approximately USD 1 billion in 2021. In comparison, one hectare of land can produce 0.12 t/year of meat, whereas the same area of water can yield 100 to 320 t/year of fish, highlighting the greater sustainability of aquaculture. Given this potential, Brazil aims to enhance its aquaculture production and meet the increasing demand for fish [2].

Among the various marine species with a commercial interest and potential for exploitation, the species of the *Centropomus* spp. genus stand out. *Centropomus* spp. is the sole genus within the predominantly marine fish family Centropomidae (Teleostei) and comprises 12 species commonly known as snooks (http://researcharchive.calacademy.org/research/ichthyology/catalog/SpeciesByFamily.asp, accessed on 19 June 2023). These species can be found in both the eastern Pacific and western Atlantic [3,4], which were separated around 1.6 to 3 million years ago due to the closure of the Panama Isthmus [5]. Among them, six species inhabit the Brazilian coasts, with *Centropomus undecimalis* (common snook, sergeant fish, or robalo) and *Centropomus parallelus* (fat snook, smallscale fat snook, little snook, or chucumite) being of particular interest for economic and sport fishing. These species have a wide distribution, ranging from southern Florida (Gulf of Mexico) to southern Brazil (Rio Grande do Sul).

These species are carnivorous, primarily feeding on fish, shrimp, and other crustaceans. However, their feeding habits exhibit some seasonal variation, as they demonstrate opportunistic predation depending on the environmental availability [6,7]. Due to the high quality and organoleptic attributes of their meat, these species command high prices in both domestic and foreign markets. As a result, they are exploited through sportive, artisanal, and professional fishing. The lack of comprehensive catch data poses challenges to their conservation [8,9,10,11,12].

*C. undecimalis*, reaching sizes of up to 140 cm and weighing up to 22 kg [13], is one of the largest species within the genus. It has been actively included in several aquaculture programs along the Atlantic Ocean’s coastal areas [4,14,15]. On the other hand, *C. parallelus* can grow up to 75 cm and weigh up to 4 kg, with females generally being larger than males of the same age [16]. Snook species are typically commercially harvested at sizes ranging from 0.5 to 1.0 kg [16].

To enhance production efficiency and profitability in aquaculture, a diverse range of genetic resources is necessary. The research focused on genomics, genetics, and breeding in aquaculture aims to improve production efficiency, sustainability, product quality, food safety, consumer protection, and overall profitability, ultimately benefiting consumers [17]. Therefore, understanding the genetic diversity of species is essential for ensuring the sustainability of future populations within their geographic range, despite habitat alterations, as well as for captive breeding programs [18,19,20].

Population genetics encompasses the study of allelic distribution and changes resulting from natural selection, mutation, migration, gene flow, geographic distribution, and genetic drift [21]. Loss of genetic variability can impact fish adaptation and survival. Therefore, understanding the population structure and genetic diversity of common and fat snook is crucial for fish production and conservation efforts. Proper management of the species relies on recognizing the genetic differences between natural reserves and different fish farming systems [22]. In modern genetic analysis, various tools are available, with genome analysis and specific genomic regions being particularly prominent. Among these tools, microsatellites have gained significant attention. Microsatellite markers, also known as Short Tandem Repeats (STR), consist of a non-transcribed, variable number of tandem repeat sequences ranging from 90 to 350 base pairs in specific locations within the genome. They exhibit co-dominance and are typically highly polymorphic, making them valuable for studying genetic diversity and quantitative trait loci [23,24,25,26].

In this study, we employed microsatellite markers to assess the genetic variability and characterization of *C. undecimalis* and *C. parallelus* populations. Our aim was to determine if these species are geographically connected. We utilized 10 microsatellite loci as genetic markers, which allowed us to gather valuable data for management, sustainability, and conservation strategies for these species. Furthermore, the genetic information obtained in this study can serve as a foundation for future breeding programs focused on snook species.

## 2. Materials and Methods

### 2.1. Sample Collection and DNA Extraction

Samples of *C. undecimalis* and *C. parallelus* specimens were collected from artisanal fisheries in the Santa Catarina to São Paulo region of Brazil (Table 1). Specifically, 42 samples of *C. parallelus* were collected, with 29 samples originating from Balneário de Penha–SC (pSC) and 13 samples from Guarujá–SP (pSP). In addition, 66 samples of *C. undecimalis* were collected, including 18 samples from Balneário de Penha–SC (uSC), 8 samples from Guarujá–SP (uSP), and 40 captive samples from CERES/UDESC (College of Southern Region/Santa Catarina State University) and UFSC (Federal University of Santa Catarina)-LAPMAR (Marine Fish Laboratory) (uCap).

Biological tissues from the animals (dorsal or caudal fin samples or muscle tissue) were collected for DNA extraction. The collected samples were stored in 1.5 mL microtubes containing 100% ethanol and kept in a freezer at −20 °C in the Laboratory of Genetics and Molecular Biology at CERES-UDESC in Laguna, SC, Brazil.

### 2.2. DNA Extraction

DNA extraction was carried out using the phenol/chloroform protocol as described by Sambrook et al. [27]. Approximately 30 mg of tissue was transferred to 1.5 mL microtubes containing 500 μL of digestion solution (50 mM Tris-HCl, 50 mM EDTA, 100 mM NaCl, 1% SDS, pH 8.0). Then, 10 μL of SDS and 20 μL of Proteinase K (20 mg/mL) were added to the tube. The tubes were gently shaken by hand for 15 s, and the solution was then incubated at 55 °C for 2 h. After incubation, 500 μL of phenol/chloroform/isoamyl alcohol was added to the tube. The mixture was then centrifuged at 12,000 rpm for 5 min. The supernatant was carefully transferred to a new tube, and 500 μL of ice-cold absolute ethanol was added. The sample was incubated overnight at −20 °C to allow DNA precipitation. Subsequently, the sample was centrifuged again for 5 min at 12,000 rpm, the supernatant was discarded, and the DNA pellet was washed with 70% ethanol. The pellet was then centrifuged again at 12,000 rpm for 15 min. After discarding the supernatant, the DNA pellet was allowed to dry in a 50 °C oven for 15 min. Finally, the DNA was resuspended in 60 μL of autoclaved distilled water and stored at −20 °C. The quantification and verification of the DNA concentration were performed using 1% agarose gel electrophoresis with a quantification marker.

### 2.3. DNA Amplification by PCR

PCR reactions were performed to amplify the loci using 10 STR markers: Cun01, Cun05B, Cun08, Cun09, Cun10A, Cun11, Cun14, Cun16, Cun21B and Cun22. The primer pairs for these markers were described by Seyoum et al. [28]. The PCR reaction mixtures were prepared with the following reagents and concentrations to a final volume of 25 μL: 50 ng of DNA, 2.5 μL of 10× buffer (100 mM Tris-HCl, pH 8.3, 500 mM KCl), 1 μL of 15 mM MgCl_2_, 2 μL of the dNTPs mixture (0.2 mM each of dATP, dCTP, dGTP, and dTTP), 1 μM of each primer (20 μg/μL), 1.25 μL of Taq Polymerase enzyme (Invitrogen), and H_2_O to complete the final volume. The PCR reactions were carried out in a thermal cycler with an initial denaturation step at 94 °C for 5 min, followed by 35 cycles of denaturation at 94 °C for 45 s, annealing at a temperature of 58–60 °C (depending on the primer pair) for 90 s, and extension at 72 °C for 7 min.

### 2.4. Data Analysis

The sequences obtained were analyzed using the GenAlEx 6.5 program [29] to calculate allele frequency, observed and expected heterozygosity, chi-square, and Hardy–Weinberg equilibrium. For the visual assessment of between-population differentiation, we conducted a principal component analysis (PCA) using the adegenet [30] and ggplot2 [31] packages in the R environment (R Development Core Team 2018). To estimate the diversity of each species in each sampling locality, we used the observed heterozygosity (HO), expected heterozygosity (HE), and allelic richness (Ar). In addition, inbreeding coefficients (FIS) for the populations within each sampling locality were calculated using the diveRsity [32] and PopGenKit [33] packages. Confidence intervals were obtained using 10,000 bootstrap replicates.

## 3. Results and Discussion

The microsatellite loci used in this study exhibited high polymorphism, allowing for the assessment of genetic variability in the populations of *C. undecimalis* and *C. parallelus*. Microsatellite markers have been widely used in fish species, as they exhibit variable numbers of alleles per locus. These markers are abundant in the genomes of higher organisms, including humans [34,35]. In the case of *C. undecimalis*, microsatellite loci were previously described by Seyoum et al. [28], and a study by Hernández-Vidal et al. [36] also utilized these markers. However, no previous study has been conducted using microsatellite markers in *C. parallelus*. The length of the alleles in this study ranged from 80 bp for locus Cun21B to 293 bp for locus Cun09 in *C. undecimalis*. In the study by Seyoum et al. [28], the variation ranged from 100 bp for locus Cun21B to 243 bp for locus Cun14.

The number of alleles varied among the three populations of *C. undecimalis*. In the uSC population, the loci Cun08 and Cun11 exhibited the highest number of alleles (22), while locus Cun05B had the lowest number of alleles (7). In the uSP population, locus Cun12 had the highest number of alleles (12), while locus Cun05B had the lowest number of alleles (3). In the uCap population, locus Cun01 had the highest number of alleles (32), while locus Cun05B had the lowest number of alleles (13). For *C. parallelus* in the pSC population, locus Cun08 had the highest number of alleles (24), while locus Cun05B had the lowest number of alleles (4). In the pSP population, locus Cun14 exhibited the highest number of alleles (15), while locus Cun16 had the lowest number of alleles (6).

Although the loci with the highest number of alleles varied, locus Cun05B consistently exhibited the lowest number of alleles (ranging from 3 to 13) in all populations of *C. parallelus* and *C. undecimalis*. A high number of different alleles per locus is indicative of high polymorphism. However, it is important to consider the sample size of each group studied, as this can strongly influence the number of alleles detected [37,38]. For instance, in the uCap population, which had the largest sample size, the highest number of alleles was observed across all loci.

In addition to allele number, other parameters need to be considered in population genetics analysis. Ho (observed heterozygosity) and He (expected heterozygosity) are reliable parameters. Notably, locus Cun05B consistently exhibited the lowest Ho value. In the uSP population, this same locus had a Ho value of 0.5, while all other loci had a Ho value of 1.

The presence of null alleles in population genetics can affect estimates of genetic variability and genetic distances [39]. Despite the high He index observed for all loci, suggesting the absence of null alleles in the populations of *C. parallelus* and *C. undecimalis* analyzed in this study, some null alleles were detected. In *C. undecimalis*, null alleles were observed in loci Cun5B, Cun16, and Cun21B in the uSP population; loci Cun14, Cun21B, and Cun22 in the uSC population; and loci Cun10A, Cun11, Cun14, and Cun22 in the uCap population. In *C. parallelus*, null alleles were detected in loci Cun10A, Cun11, and Cun16 in the pSC population; and loci Cun14, Cun21B, and Cun22 in the pSP population.

In the current study, the analysis of genotypic and genotypic differentiation based on observed allele and genotype frequencies revealed possible genetic differentiation among the populations analyzed. Studies on population differentiation in fish have been conducted to identify and manage distinct genetic pools. Population differentiation can manifest in various forms, ranging from genetically distinct populations to panmictic populations. The conservation units are typically represented by genetically homogeneous groups of individuals, such as local populations [40].

Regarding the populations of *C. undecimalis*, in the fSP population, the observed heterozygosity (Ho) ranged from 0.5 to 1, with an average of 0.95, while the expected heterozygosity (He) ranged from 0.8 to 0.91, with an average of 0.82. In the fSC population, Ho ranged from 0.39 to 1, with an average of 0.89, while He ranged from 0.9 to 0.94, with an average of 0.89. In the fCap population, Ho ranged from 0.68 to 1, with an average of 0.93, while He ranged from 0.85 to 0.95, with an average of 0.92. Regarding the populations of *C. paralellus*, in the pSC population the HO ranged from 1 to 0.55 with an average of 0.86, while the He ranged from 0.9 to 0.94 with an average of 0.89. In the pSP population, the Ho ranged from 1 to 0.77 with an average of 0.95, whereas the He ranged from 0.9 to 0.85 with a mean of 0.84.

**Figure 1:**
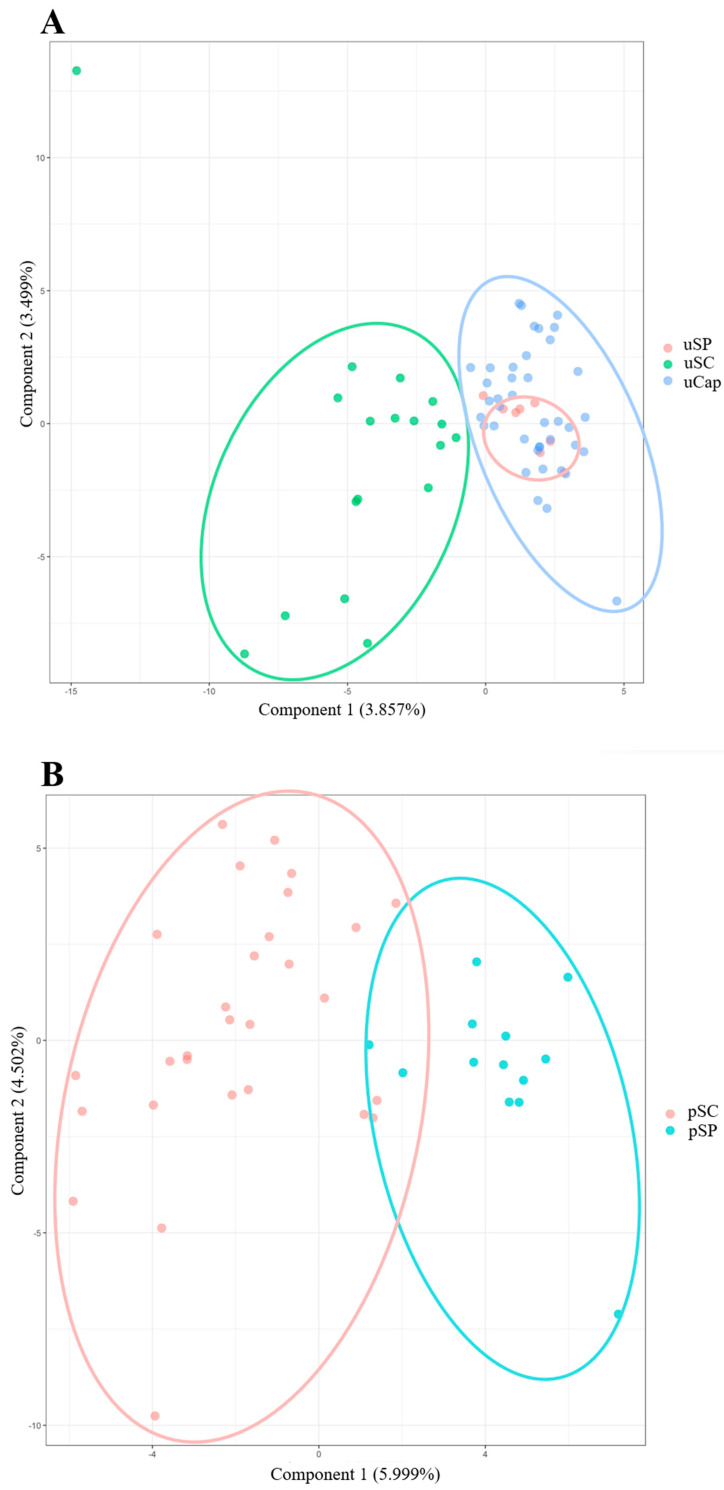
Dispersion plots of the first (PC1) and second (PC2) principal components based on the analysis of the individual *C. undecimalis* (**A**) and *C. parallelus* (**B**) using 10 microsatellite loci. The ellipses, shown in different colors, represent the analyzed individuals in two different analyses. Populations are represented in the figures with the acronyms used in this study: captive *C. undecimalis* (uCap) in São Paulo (uSP) and Santa Catarina (uSC); and *C. parallelus* in São Paulo (pSP) and Santa Catarina (pSC).

The coefficient of inbreeding (FIS) did not show negative values, indicating that there is no excess of heterozygotes in these populations. FIS varied among all loci and populations of both species, as shown in Table 2 and Table 3. The FST (fixation index) value of 0.0376 with a confidence interval of (0.0250–0.0532) indicated a low level of genetic differentiation among the *C. undecimalis* populations, according to Wright [41]. The principal component analysis (PCA) data revealed genetic distinction between populations, particularly between the pSC and pSP populations of *C. parallelus*, and the uSC and uSP populations of *C. undecimalis*. However, the uCap population showed a closer genetic relationship to the uSP population than to the uSC population (Figure 1).

The loci (Cun01, Cun05B, Cun08, Cun09, Cun10A, Cun11, Cun14, Cun16, Cun21B, and Cun22) did not conform to Hardy–Weinberg equilibrium across populations and species [42]. The differences between Ho and He values indicate the presence of selection forces, whether natural or sexual. There was no evidence of linkage disequilibrium between the loci, suggesting that they are genetically independent.

The capture of these species is associated with migratory movements to freshwater ecosystems and spawning along the coast. The environmental differences found along the coast have an ecological and evolutionary impact, favoring genetic differentiation. Surprisingly, there have been no studies comparing the species and populations of *C. undecimalis* and *C. parallelus* between the coastal regions of the Brazilian states of São Paulo and Santa Catarina [43,44,45].

Although the scientific literature lacks sources on these two species, especially regarding their genetics, some considerations can be made based on our data. In summary, our data demonstrate that these populations exhibit high genetic variability, including the recently domesticated population of *C. parallelus*. The populations also showed distinct allelic frequencies for certain alleles, indicating the presence of genetically consolidated populations. Genetically consolidated populations refer to groups of organisms of the same species that share similar genetic characteristics and have a shared evolutionary history [39]. The figure provided in the study indicates some convergence points between populations of *C. parallelus*, suggesting the possibility of reproductive contact. Furthermore, the figure shows that the domesticated population of *C. undecimalis* is more closely related to the population of São Paulo than to Santa Catarina.

The initial hypothesis of this study was that the populations of both species in Santa Catarina and São Paulo would exhibit little difference, given the relative proximity of these states in Brazil. However, the results of this study contradicted this hypothesis. The findings clearly demonstrate that there is no intense reproductive contact between these populations, indicating a significant degree of genetic differentiation. This is particularly interesting considering that these species have a wide distribution, spanning from the south of Florida (Gulf of Mexico) to the south of Brazil (Rio Grande do Sul).

The observed genetic differentiation suggests that factors such as geographic barriers or local environmental conditions may be contributing to the distinct genetic profiles of these populations. Further studies involving other populations along the Brazilian coast could provide additional insights and help identify which populations are more closely correlated.

It is important to note that low genetic variability is a concerning sign for the conservation of a species [29]. Therefore, ongoing studies that focus on the genetic characterization and assessment of these populations are essential. These studies contribute to our understanding of the genetic diversity and structure of the species, which in turn helps inform management and conservation efforts. By identifying and evaluating the genetic variability of these populations, we can gain valuable insights into their potential risks and implement appropriate conservation measures.

In summary, this study highlights the importance of genetic studies in assessing the populations of *Centropomus undecimalis* and *Centropomus parallelus*. The results shed light on the genetic differentiation between populations and emphasize the need for further research to understand the underlying factors driving these patterns. Such knowledge is crucial for the effective management and conservation of these species, ultimately ensuring their long-term survival and sustainability.

## 4. Conclusions

The use of microsatellite markers proved to be highly effective in studying the genetic variability of *Centropomus parallelus* and *Centropomus undecimalis* populations, providing valuable data on their genetic structuring. This information is essential for the management and conservation of these species. Moreover, the development of ten polymorphic microsatellite loci for both species opens possibilities for future studies and the potential for heterologous amplification. Although neither species is currently listed as endangered, the genetic differences observed among the studied populations highlight the importance of considering genetic diversity even within species. The Centropomidae species, including *C. parallelus* and *C. undecimalis*, hold significant economic and recreational value, particularly in the context of sport fishing. The findings of this study can serve as a foundation for future breeding programs and contribute to the development of tools for fish farming. It is worth noting that this study represents the first genetic characterization of populations of *C. parallelus* and *C. undecimalis* in Brazil, their primary habitat. This genetic characterization plays a crucial role in monitoring the species for conservation purposes, particularly in relation to genetic variability. The data obtained not only contribute to the understanding of the reproductive nature of these species but also shed light on their gene flow dynamics. Overall, this genetic study provides valuable insights into the populations of *C. parallelus* and *C. undecimalis*, offering a foundation for conservation efforts and informed management strategies. By understanding the genetic variability and structure of these species, we can implement effective measures to ensure their long-term survival and contribute to the sustainable use of their resources.

## Figures and Tables

**Table 1:** Relationship among the number of samples per species, place of origin and their respective abbreviations evaluated in this study.

Specie	Sample Code	Number (n) of Samples Collected	Collection Point	
			Federal State	County or Region
*C. parallelus*	pSC	29	Santa Catarina	Balneário de Penha
	pSP	13	São Paulo	Guarujá
*C. undecimalis*	uCap	40	Santa Catarina	Captive *
	uSC	18	Santa Catarina	Balneário de Penha
	uSP	8	São Paulo	Guarujá

* Captive from CERES/UDESC and UFSC-LAPMAR (Marine Fish Laboratory).

**Table 2:** The measure of the genetic structure of *Centropomus udecimalis* and *Centropomus paralellus* F-statistics. Fst is the proportion of the total genetic variance contained in a subpopulation and Fis (inbreeding coefficient) is the proportion of the variance in the subpopulation contained in an individual.

Specie	Parameters	Mean IC	Lower IC	Higher IC
*C. udecimalis*	Fst	0.0376	0.0250	0.0532
	Fis	−0.0021	−0.0262	0.0202
*C. paralellus*	Fst	0.0592	0.0368	0.0832
	Fis	0.0020	−0.0263	0.0308

**Table 3:** The measure of genetic structure of the populations of *Centropomus udecimalis* and *Centropomus paralellus*. Fis (inbreeding coefficient) is the proportion of the variance in the subpopulation contained in an individual and AR allele designations represent the number of repeats.

Polpulations	Mean_AR	Lower Bound_AR	Higher Bound_AR	Mean_Fis	Lower Bound_Fis	Higher Bound_Fis
uSP	6.58	5.2	7.9	−0.1537	−0.2618	−0.0992
uSC	8.9	6.7	10.6	−0.0049	−0.0655	0.0455
uCap	9.9	8	11.3	−0.0113	−0.0388	0.0142
pSC	10.87	9.1	12.4	0.0275	−0.0086	0.0649
pSP	8.17	6.9	9.3	−0.1404	−0.1949	−0.1016

## Data Availability

The data in this study were not published in any database since no high-throughput sequencing data was generated.
